# The impact of ventilation mode on systemic inflammation and organ dysfunction in a porcine model of abdominal sepsis-associated acute respiratory distress syndrome

**DOI:** 10.1186/s40635-026-00985-3

**Published:** 2026-10-10

**Authors:** James G. Williams, Michaela Kollisch-Singule, Nirmeen Elmadany, Nader M. Habashi, Gary F. Nieman, Brian M. Varisco

**Affiliations:** 1 Division of Critical Care Medicine and Department of Pediatrics, University of Arkansas for Medical Sciences, 13 Children’s Way, Slot 512-36, Little Rock, 72205 AR USA; 2 Department of Pediatric Surgery, University of Arkansas for Medical Sciences, Little Rock, 72205 AR USA; 3 Adams Cowley Shock Trauma Center, University of Maryland Medical Center, Baltimore, 21201 MD USA; 4 Department of Surgery, SUNY Upstate Medical University, Syracuse, 13210 NY USA

**Keywords:** Sepsis, Acute respiratory distress syndrome (ARDS), Systems biology, Proteomics, Critical care

## Abstract

**Introduction:**

Sepsis commonly causes acute respiratory distress syndrome (ARDS), and immune hyperactivation may be a causal link. Ventilator-induced lung injury (VILI) is common in ARDS, and VILI may potentiate organ dysfunction in severe sepsis. We used a porcine model of abdominal sepsis-associated ARDS to test whether ventilation strategy could impact systemic inflammation and organ dysfunction.

**Methods:**

Female Yorkshire pigs (6–8 months) underwent ischemia–reperfusion (I/R) injury of the small bowel and fecal clot implantation or sham laparotomy. The abdominal sepsis animals were ventilated with a volume-controlled (VC) mode using the ARDS-Net low tidal volume (LVt) method to set and adjust the mode, and applied at two time points. Late LVt, was applied once PaO_2_/FiO_2_ was ≤300, and Early LVt was applied immediately following injury. The third lung protective group used the airway pressure release ventilation (APRV) mode set and adjusted with the time-controlled adaptive ventilation (TCAV) method, also applied immediately after injury. Animals were supported for 48 h using standardized resuscitation protocols and euthanized. Physiologic variables, clinical lab data, lung histology, and plasma proteome were assessed.

**Results:**

Sham animals showed no evidence of sepsis or ARDS, whereas all experimental animals developed septic shock, requiring fluid resuscitation and vasopressors. Both LVt groups developed ARDS as measured by PaO_2_/FiO_2_ ratio by the study end (Late LVt: 129.9±16.0; Early LVt: 205.2±24.2), while the Early APRV group did not (429.4±59.4). The Sham and experimental groups all had similar proteomes at baseline. The proteomes diverged over time and ended in three distinct groups: Sham, Late LVt/Early LVt, and Early APRV. The most significantly differentially expressed proteins distinguishing the groups were involved in the immune and inflammatory response, with APRV having the lowest inflammatory signature.

**Conclusion:**

In a porcine model of abdominal septic shock we found that animals ventilated with a higher mean airway pressure strategy (APRV) had decreased plasma proteomic signatures of inflammation compared to those ventilated with a conventional low tidal volume strategy. Our findings are hypothesis generating and not directly translatable to the clinical setting. Future studies should focus on further elucidating a link between VILI and its effect on systemic inflammation and organ dysfunction.

**Supplementary Information:**

The online version contains supplementary material available at https://doi.org/10.1186/s40635-026-00985-3.

## Background

Despite advances in supportive care, sepsis remains a critical health burden and a leading cause of death among hospitalized and critically ill patients. According to the Global Burden of Disease data, approximately 49 million cases of sepsis are estimated to occur worldwide each year [1]. Every year in the United States, 1.7 million adults develop sepsis, with approximately 201,092 deaths in 2019 linked to sepsis [2]. Sepsis arises from a dysregulated host immune response to infection, leading to tissue damage, organ failure, and potentially death [3]. The pathophysiology of the dysregulated host immune response is complex and heterogeneous [4]. This leads to subgroups of sepsis patients that have vastly different degrees of organ failure, morbidity, and mortality despite similar clinical characteristics and infectious sources [5, 6]. The biological basis underlying these subgroups is not entirely understood and represents a knowledge gap that hinders the development of new therapies [7].

Acute respiratory distress syndrome (ARDS) is defined by hypoxemia and radiographic lung opacities that appear within seven days of a triggering event. It is a common comorbidity in patients with sepsis [8–10] and septic patients who develop ARDS have a higher risk of mortality compared to those without ARDS [10, 11]. Like sepsis, ARDS arises from a maladaptive immune response to an insult resulting in non-cardiogenic pulmonary edema, alveolar inflammation, and alveolar damage [9]. Management in ARDS primarily consists of supportive care, mechanical ventilation, and treatment of any underlying cause [12–14]. Recent work suggests that supportive strategies may affect outcomes differently across subgroups of ARDS patients [15, 16]. Many patients require mechanical ventilation for support, yet the ventilator itself may cause unintended secondary ventilator-induced lung injury (VILI) by producing mechanical forces that further amplify inflammation [17]. Strategies to mitigate VILI include protective ventilation with lower tidal volumes (Vt) and higher mean airway pressures, which have been shown to attenuate pro-inflammatory cytokine expression [12, 18]. Since VILI increases inflammatory cytokines that can amplify the sepsis-induced dysregulated immune response [19–21], and the degree of inflammation influences outcomes [6], augmented inflammation from VILI could increase sepsis-related mortality.

The overall goal of this study was to use our previously described porcine model of abdominal sepsis [22] to explore how the strategy of ventilation alters the degree of systemic inflammation by sampling the plasma proteome of animals treated with conventional low Vt (LVt) ventilation, airway pressure release ventilation (APRV) using the time controlled adaptive ventilation (TCAV) method [23], or injurious mechanical ventilation (Late LVt group). Our primary physiologic outcomes of interest were P/F ratio, creatinine, and sequential organ failure assessment (SOFA) score at the end of the experiment. We then sought to explore how the plasma proteome and specifically, the degree of inflammation correlated with ventilator strategy and our physiologic outcomes. Since both sepsis and VILI are inflammatory processes that might potentiate one another, we hypothesized that lung-protective ventilation modes would be associated with less systemic inflammation and organ dysfunction than less protective strategies.

## Methods

### Animal care and ethical standards

All experiments were performed in accordance with National Institute of Health (NIH) guidelines in the use of laboratory animals and approved by the SUNY Upstate Medical University Institutional Animal Care and Use Committee (IACUC) protocol numbers 243 and 332. Details of animal housing, husbandry, and monitoring are provided in the Supplementary Methods.

### Sample size

The goal of this study was to provide evidence that our hypothesis regarding the effects of mechanical ventilation on systemic inflammation during sepsis was plausible. As such, we did not attempt to power our study to demonstrate causality or a definitive relationship between ventilator strategy and systemic inflammation. Given the significant logistical barriers to conducting 48 h large animal experiments, it was necessary to limit the numbers of animals in each group. Thus, we decided to include 4 animals in each of our experimental groups and five animals in the sham group.

### Surgical model

Female Yorkshire pigs (30–39kg) were used to minimize variability within the model. They were anesthetized using a continuous infusion of intravenous ketamine (90 mg/kg) and xylazine (10 mg/kg) to maintain a surgical plane of anesthesia. Under sterile conditions, a tracheostomy was performed with a 7.5 mm endotracheal tube (Harvard Apparatus) and connected to a mechanical ventilator (Dräger Evita Infinity V500, Lübeck, Germany). A tracheostomy was selected to ensure standardized tip placement among animals to avoid esophageal and mainstem intubation. Peri-procedural ventilator settings were set in a volume control (VC) mode with baseline Vt of 10 mL/kg, respiratory rate (RR) of 12 breaths/min, positive end-expiratory pressure (PEEP) of 5 cmH_2_O, and a fraction of inspired oxygen (FiO_2_) of 100%. A central venous catheter was placed for the administration of fluids and medications. An arterial catheter (Edwards Lifesciences, Irvine) was placed in the femoral artery and used for arterial blood pressure monitoring and blood gas (ABG) measurements. The bladder was catheterized for intra-abdominal pressure (IAP) monitoring (ConvaTec Inc., NJ) and to measure urine output. Baseline (BL) measurements were taken after surgical preparation and before injury.

In the experimental groups, extrapulmonary lung injury was induced using a previously established double-hit model of ischemia–reperfusion and fecal peritonitis [22, 24, 25]. Briefly, the superior mesenteric artery was clamped for 30 min and then released to induce intestinal ischemia and subsequent reperfusion injury. Peritoneal sepsis was induced by creating an enterotomy in the cecum and mixing a fecal clot (1mL/kg feces with 2mL/kg blood) to be implanted into the peritoneal cavity. Time Zero (T0) measurements were taken immediately after the induction of the double-hit injury and upon the closure of the abdomen. Sham animals underwent sham laparotomy followed by post-operative care as described below.

### Resuscitative protocol

Intravenous (IV) fluid resuscitation and vasopressor administration were guided by the Surviving Sepsis campaign guidelines [26]. Antibiotic selection was based on likely pathogenic organisms from prior experiments. Animals received continuous maintenance IV fluid resuscitation and boluses as needed with Lactated Ringers to maintain a mean arterial pressure (MAP) greater than 65 mmHg. Continuous norepinephrine infusion was initiated when the animal was no longer fluid-responsive as determined by persistent hypotension (MAP < 65 mmHg) following two consecutive 20 mL/kg fluid boluses. Norepinephrine was titrated as needed to maintain a MAP > 65 mmHg to a maximum of 20 mcg/min. Vasopressin was initiated at 0.04 U/min when the norepinephrine rate reached 10mcg/min. Epinephrine was initiated and adjusted up to 10 mcg/min when the norepinephrine dose of 20 mcg/min was insufficient to maintain normotension. Rocuronium was administered whenever spontaneous respiratory effort was present to standardize animals across groups.

### Post-surgical ventilator management

The strategy used to determine initial settings and subsequent ventilator titration are described by group as follows (Supplemental Table 1):

**Sham Group:** Animals were maintained in a VC mode with Vt 10 mL/kg, PEEP 5 cmH_2_O, and RR 12 breaths/minute. Only FiO_2_ was modified as needed to maintain oxygen saturation (SpO_2_) at or above 90%.

**Late LVt Group (Late LVt):** This group was intended to simulate VILI in patients with severe ARDS that are unable to achieve the usual goals set for lung protective mechanical ventilation. Animals were continued in a VC mode of ventilation with Vt 10 mL/kg and PEEP 5 cmH_2_O, RR of 12 breaths/minute, and FiO_2_ 21%. They were transitioned to a LVt strategy of 6 ml/kg and PEEP of 5 cmH_2_O only upon reaching clinical ARDS per the Berlin definition (PaO_2_/FiO_2_ < 300 mmHg) [27]. Subsequently, all ventilator adjustments were made following the ARDSnet protocol with PEEP and FiO_2_ titrated according to SpO_2_ and PaO_2_ following the low PEEP:high FiO_2_ strategy [12]. RR was titrated to maintain a pH > 7.2 and Vt was titrated as needed to keep a plateau pressure < 30 cmH_2_O or until it reached a minimum of 4 ml/kg as recommended in the ARDSnet protocol.

**Early LVt Group (Early LVt):** This group was intended to simulate the usual clinical care of intubated patients with ARDS. Animals were transitioned from the baseline settings to LVt settings of Vt 6 mL/kg, PEEP of 5 cmH_2_O, RR 12 breaths/minute, and FiO_2_ 21% immediately after surgical closure at T0. All ventilator adjustments were made in accordance with the low PEEP:high FiO_2_ table from the ARDSnet protocol [12], as described above. RR was titrated to maintain a pH > 7.2 and Vt was titrated as needed to keep a plateau pressure < 30 cmH_2_O or until it reached a minimum of 4 ml/kg as recommended in the ARDSnet protocol.

**Early APRV Group (Early APRV):** This group was intended to explore the effects of APRV as an alternative lung protective strategy to conventional ventilation with low Vt. APRV is a time cycled, pressure-controlled mode that is defined by four parameters: the value of the constant pressure given through the airway opening during inspiration (P_High_), the duration of P_High_ (T_High_), the constant pressure applied during expiration (P_Low_), and the duration of P_Low_ (T_Low_). A previously described TCAV method that has demonstrated lung protection [24, 25, 28] was used to set and adjust the APRV mode [29]. Briefly, animals were ventilated at P_High_ set at the plateau pressure (Pplat) obtained during the VC setting used for BL measurements (16–22 cmH_2_O) for a T_High_ of 4.0–4.5 s, which was set to occupy approximately 90% of the total ventilator cycle time. The P_Low_ was set at 0 cmH_2_O to minimize expiratory resistance and maximize the peak expiratory flow rate (PEFR). The T_Low_ was set to achieve a ratio of the End Expiratory Flow Rate (EEFR) to Peak Expiratory Flow Rate (EEFR:PEFR) of 75%, which was between 0.32–0.37 s. P_High_, P_Low_, T_High_, T_Low_, and FiO_2_ were titrated throughout the study according to pulmonary parameters, PaO_2,_ and PaCO_2_.

### Physiologic measurements, SOFA score, and specimen collection

Pulmonary variables were recorded from the ventilator every hour. Hemodynamics were continuously monitored (Intellivue MP-90, Phillips Healthcare, Irvine, CA) using Edwards transducers (Pressure Monitoring Kit, Edwards Lifesciences, Irvine, CA). The ABGs were measured every 1–3 h with a Roche blood gas analyzer (Cobas b221, Basel, Switzerland). Sequential Organ Failure Assessment (SOFA) scores were calculated as previously described [30] with the GCS of all pigs designated 3T as all animals were intubated and muscle relaxed. Plasma samples were drawn every 12 h for electrolyte and cytokine analyses. Clinical pathology laboratories were performed at 24-h intervals by the Upstate Medical University pathology laboratory facility. Plasma samples for proteomic analyses were collected at baseline, 6, 12, 24, 36, and 48 h in the Early LVt, Late LVt, and Early APRV groups. Plasma samples for proteomic analyses were collected at baseline, 24 h, and 36 h in the sham group.

### Necropsy

After 48 h, resuscitation was terminated, the animals were euthanized, and a necropsy was performed. The lungs were inflated to 25 cmH_2_O using stepwise increases in PEEP to standardize lung volume and then removed. The left lung was filled with 10% formalin to a height of 25 cm, clamped, and submerged in formalin. Specimens were then embedded in paraffin, sectioned, and stained with hematoxylin and eosin.

### Quantitative histology

Lung histology of the experimental groups was compared to that of a healthy, euthanized control animal (termed "Naïve"). The quantitative histological assessment of the lung was based on image analysis of 120 photomicrographs (10 per animal). These photomicrographs were taken at high dry magnification using a validated, blinded, and systematic sampling protocol [22]. Each photomicrograph was scored using a 4-point scale for each of the 5 parameters: atelectasis, fibrinous deposits and blood in air space, vessel congestion, and alveolar wall thickness.

### Biostatistics

Continuous pulmonary and cardiovascular physiologic data were analyzed using the Kruskal–Wallis test. If the Kruskal–Wallis test was significant, Dunn’s test with Bonferroni correction was performed to test for pairwise differences between groups. Nonparametric testing was chosen given the increased risk of Type 1 error when using parametric tests to evaluate data when the sample size is small. Differences in histopathology, SOFA score, and creatinine were evaluated using a repeated measures analysis of variance (ANOVA) test with post hoc Tukey’s test to determine between-group differences. For all tests, a p-value of < 0.05 was considered statistically significant.

### Proteomics

The plasma was obtained from previously published studies [18, 22, 25]. Plasma specimens were sent to the IDeA National Resource for Quantitative Proteomics at the University of Arkansas for Medical Sciences (UAMS) for processing and analysis. Proteins were reduced and alkylated before digestion with sequencing-grade modified porcine trypsin (Promega) using S-Trap columns (Protifi). Tryptic peptides were then separated by reverse phase XSelect CSH C18 2.5 μm resin (Waters) on an in-line 150 × 0.075 mm column using an UltiMate 3000 RSLCnano system (Thermo). Peptides were then eluted using a 60 min gradient from 98:2 to 65:35 buffer A:B ratio (buffer A = 0.1% formic acid, 0.5% acetonitrile; buffer B = 0.1% formic acid, and 99.9% acetonitrile). Eluted peptides were ionized by electrospray (2.2 kV) followed by mass spectrometric analysis on an Orbitrap Exploris 480 mass spectrometer (Thermo). To assemble a chromatogram library, 6 gas-phase fractions were acquired on the Orbitrap Exploris with 4 m/z DIA spectra (4 m/z precursor isolation windows at 30,000 resolution, normalized automatic gain control (AGC) target 100%, maximum inject time 66 ms) using a staggered window pattern from narrow mass ranges using optimized window placements. Precursor spectra were acquired after each DIA duty cycle, spanning the m/z range of the gas-phase fraction (i.e., 496–602 m/z, 60 000 resolution, normalized AGC target 100%, maximum injection time 50 ms). For wide-window acquisitions, the Orbitrap Exploris was configured to acquire a precursor scan (385–1015 m/z, 60 000 resolution, normalized AGC target 100%, maximum injection time 50 ms) followed by 50× 12 m/z DIA spectra (12 m/z precursor isolation windows at 15 000 resolution, normalized AGC target 100%, maximum injection time 33 ms) using a staggered window pattern with optimized window placements. Precursor spectra were acquired after each DIA duty cycle. The mass spectrometry proteomics data have been deposited to the Proteome X change Consortium via the PRIDE [31] partner repository with the dataset identifier PXD029576.

### Protein identification and normalization

Bioinformatic analyses were performed in R [32] with the packages noted below. Following data acquisition, data were searched using an empirically corrected library, and a quantitative analysis was performed to obtain a comprehensive proteomic profile. Proteins were identified and quantified using EncyclopeDIA [33] and visualized with Scaffold DIA using 1% false discovery thresholds at both the protein and peptide level. Protein-specific intensity values were assessed for quality using an in-house ProteiNorm app, a tool for systematic evaluation of normalization methods, imputation of missing values, and comparison of multiple differential abundance methods [34]. Normalization methods evaluated included log2 normalization (Log2), median normalization (Median), mean normalization (Mean), variance stabilizing normalization, quantile normalization, cyclic loess normalization, global robust linear regression normalization, and global intensity normalization (Global Intensity). The individual performance of each method was evaluated and verified by comparing the following metrics: total intensity, pooled intragroup coefficient of variation, pooled intragroup median absolute deviation, pooled intragroup estimate of variance, intragroup correlation, sample correlation heatmap (Pearson), and log2-ratio distributions.

### Missing values and batch correction

After normalization, proteins with greater than 50% of values missing were excluded from further analysis. For proteins with less than 50% of missing values, the missing values were imputed using the quantile regression imputation of left-censored data (QRILC) method via the imputeLCMD package (version 2.0) [35]. This method was chosen because in quantitative proteomics missing data are often attributable to proteins with concentrations below the lower limit of detection, known as missing not at random (MNAR) values. The QRILC method treats missing values as left censored and imputes them by drawing random values from a truncated normal distribution estimated from the lower tail of the observed intensity distribution for each protein. This method better preserves the left shifted character of MNAR values compared with other methods such as k-nearest neighbor imputation.

Our experiment was conducted in batches given the practical difficulties of conducting large animal experiments of this nature. To address the potential for batch effects, we performed batch correction, although this does not fully exclude the possibility of residual batch effects. The three batches were: Batch 1 (sham), Batch 2 (Early APRV), and Batch 3 (late and early LVt). To preserve biological differences while removing technical variation, batch effect parameters were estimated using only baseline samples with a baseline-anchored approach and the ComBat algorithm from the R package sva (version 3.42.0) [36]. The batch correction parameters calculated from the baseline samples were then applied to all other timepoints.

### Differential protein expression and principal component analysis

Principal component analysis (PCA) was performed on scaled protein expression data using the prcomp function in base R [32]. PCA results were visualized to assess separation patterns between experimental groups and temporal progression. Group centroids, connected by arrows, were used to illustrate proteome trajectories over time. Differential protein expression analysis was conducted using linear mixed effects models using lme4 [37] and lmerTest [38] in R. For each protein, levels were modeled as: *Expression ∼ Group x Time+(1|Animal)* where group represents the ventilation mode (Sham, early LVt, late LVt, or Early APRV), time is the numeric time point (0, 6, 12, 24, 36, and 48 h), and animal serves as a random effect to account for repeated measures within subjects. Models were fitted using restricted maximum likelihood with the bobyqa optimizer and extended iteration limits to ensure convergence. Model convergence was assessed for each protein, and proteins with singular fit warnings or convergence failures were flagged. Statistical significance was determined using type III ANOVA tests to evaluate the main effects of ventilation group and time, and the interaction between group and time. P-values were adjusted for multiple comparisons using the Benjamini–Hochberg false discovery rate (FDR) correction, with statistical significance defined as an adjusted p-value < 0.05.

### Pathway enrichment analysis

Protein symbols were converted to Entrez Gene IDs using the clusterProfiler [39] package and org.Hs.eg.db annotation database, with pig proteins mapped to human orthologs for pathway analysis and compatibility. All enrichment analyses were conducted using clusterProfiler and used a p-value cutoff of 0.05, q-value cutoff of 0.2, gene set size 3–500, and Benjamini–Hochberg correction for multiple comparisons. The Reactome database was used for pathway analysis using the ReactomePA package [40]. Gene Ontology Biological Processes enrichment was also performed for timepoint specific pairwise comparisons using clusterProfiler.

### Analysis of hub proteins

Protein–protein interaction networks were constructed using the STRING database (v11.5) via the STRINGdb [41] R package. Gene symbols from significantly differentially expressed proteins (adjusted p-value < 0.05) were mapped to STRING identifiers, and interactions were retrieved with a confidence score threshold of 400. Hub genes were identified based on network topology metrics calculated using the igraph [42] R package. For each protein network, the following centrality measures were computed: degree centrality, betweenness centrality, closeness centrality, eigenvector centrality, and PageRank centrality. A composite score was calculated, and proteins were ranked accordingly. The top 15 hub proteins were then identified. To determine the regulatory direction of hub genes, log2 fold changes (log2FC) were calculated by comparing the mean protein expression levels between the experimental and sham groups. Proteins were classified as upregulated if log2FC was > 0.2, downregulated if the log2FC was < − 0.2, and unchanged if the log2FC was between − 0.2 and 0.2. Network visualizations were generated using the igraph package in R. Protein–protein interaction (PPI) networks were visualized using force-directed Fruchterman-Reingold layouts. To explore the clinical relevance of the proteins identified in the PPI networks, we selected the top five hub proteins identified in the Group Effects and Group Effects x Time models. We then correlated the expression of these proteins with lactate and SOFA score using Spearman rank correlation across all available animal timepoint observations. P-values were adjusted for multiple comparisons using the Benjamini–Hochberg method.

## Results

### ARDS incidence and ventilatory requirements

**Fig. 1:**
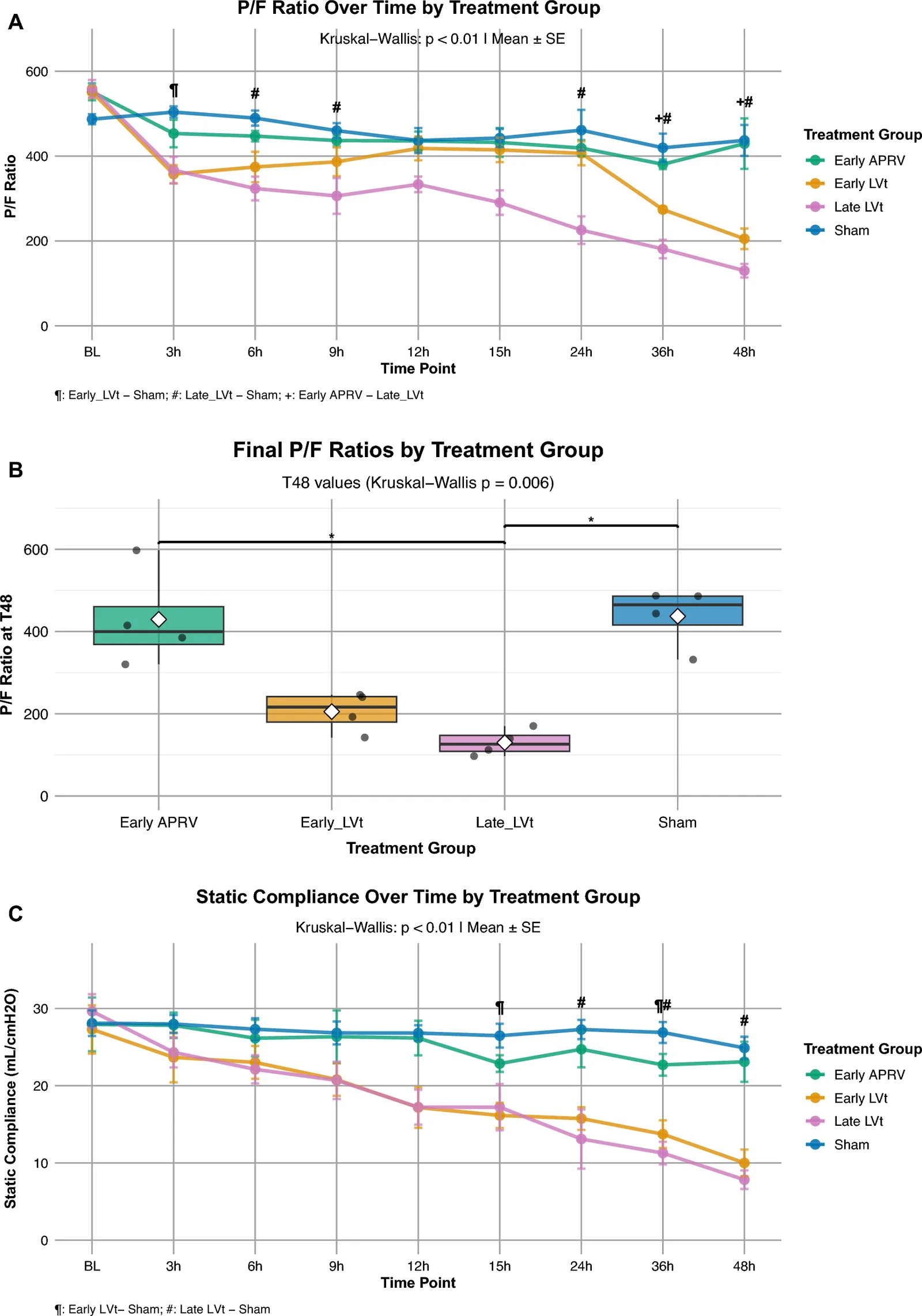
**A**) P_a_O_2_/FiO_2_ (P/F) ratio over time from baseline (BL) through the experiment end (48h) comparing Sham (no injury—blue) against three different mechanical ventilation strategies (APRV—green), Early LVt—orange), and Late LVt—pink) after ischemia/reperfusion and peritonitis injury. Data points are mean ±SE. **B**) Final P/F ratios at the end of the experiment comparing sham, APRV, Early LVt, and Late LVt. Black horizontal lines represent the median, while the diamonds represent the mean. The vertical black lines represent standard error. **C**) Static compliance (ml/cmH_2_O) over time among groups

There were four pigs each in the Early LVt, Late LVt, and APRV groups, and five pigs in the Sham group. There was a significant difference in the mean P_a_O_2_/F_i_O_2_ (P/F) ratio among the four experimental groups over time (p<0.05 among groups; Fig. 1A). The Early LVt and Late LVt groups had the most significant initial drops in P/F, followed by a steady decline. The Early and Late LVt groups met ARDS criteria with a P/F ratio < 300 by 32 ± 2 h (Early LVt) and 21 ±4 h (Late LVt). The mean P/F ratio for the Sham and Early APRV groups never dropped < 300. By the end of the study, the Late LVt group established moderate ARDS with a mean P/F ratio of 130±16, whereas the Early LVt group developed mild ARDS with a P/F ratio of 205±24 (Fig. 1B). Neither the Early APRV nor the Sham group met ARDS criteria at the end of the study, with P/F ratios of 429±59 (APRV) and 437±36 (Sham). The static compliance trended down in the Late LVt and Early LVt groups throughout the study, but remained stable in the Sham and Early APRV groups. At the end of the study, compliance was significantly reduced in the Early (10.0±1.7mL/cmH_2_O) and Late LVt (7.8±1.2mL/cmH_2_O) groups compared with the Early APRV (23.1±2.6mL/cmH_2_O) and Sham (24.9±1.5mL/cmH_2_O) groups (Fig. 1C).

The end-expiratory pressure in APRV is time-controlled (TC), as it is a function of T_Low_ and P_Low_, and is therefore distinguished as TC-PEEP. The TC-PEEP was significantly higher in the Early APRV group compared to the PEEP in the VC modes at earlier time points in the study (Supplemental Fig. 1A); however, by the end of the study, the Late LVt group had the highest PEEP (20 ± 3.3 cmH_2_O). By comparison, the Early LVt group ended with a PEEP of 7.1 ± 2.6 cmH_2_O. In the Early APRV group, the end-expiratory pressure was 18.1 ± 2.9 cmH_2_O. There was a significant difference in Pplat at the end of the study between groups, with the highest Pplat of 44.5 ± 4.2 cmH_2_O in the Late LVt group (p<0.05 among groups; Supplemental Fig. 1B) as compared with Early APRV at 34.5 ± 0.7 cmH_2_O and the Early LVt group at 24 ± 2.3 cmH_2_O. The driving pressure was not significantly different between groups at the end of the study (Supplemental Fig. 2C).

By the end of the study, the mean Vt was significantly higher in the Early APRV group at 13.2±0.4 mL/kg compared to the Early and Late LVt groups (p<0.05; Supplemental Fig. 1D). The Early LVt group completed the study with Vt of 5.2±0.5mL/kg while the Late LVt group had Vt of 5.4±0.5 mL/kg. Consequently, the LVt groups had significantly higher P_a_CO_2_ levels (Early LVt: 57.4±8.2mmHg, Late LVt: 64.3±6.2mmHg) at the end of the study compared with the Early APRV group (p<0.05; Supplemental Fig. 1E).

### Hemodynamics, SOFA score, and acute kidney injury

The MAP was controlled among the groups as animals were provided fluid resuscitation and vasopressor support to maintain a pressure > 65mmHg, and such that there was no significant difference among the four groups at any time point during the study (p>0.05; Supplemental Fig. 2A). Despite this, there was a significant difference in the lactate in the later time points of the study (Supplemental Fig. 2B). The Late LVt and Early APRV groups both had significantly higher lactate levels than the sham group, with lactate levels of 10.8 ±7.2 mmol/L (Late LVt) and 4.4 ±3.8 mmol/L (APRV), respectively. All three injury groups received vasoactive support by the end of the study, and the average time to norepinephrine initiation was shortest in the Late LVt group (15.3±5.9 h versus APRV: 19.8±7.0h and Early LVt: 29.5±10.9h), although this difference was not statistically significant (p>0.05). The cumulative dose of norepinephrine was also highest in the Late LVt group (Supplemental Fig. 2C); however, this difference was also not statistically significant (p>0.05). The intra-abdominal pressure measured via bladder catheter was significantly higher in the Late LVt and Early APRV groups compared to the sham group at specific time points later in the experiment (p<0.05; Supplemental Fig. 2D).

**Fig. 2:**
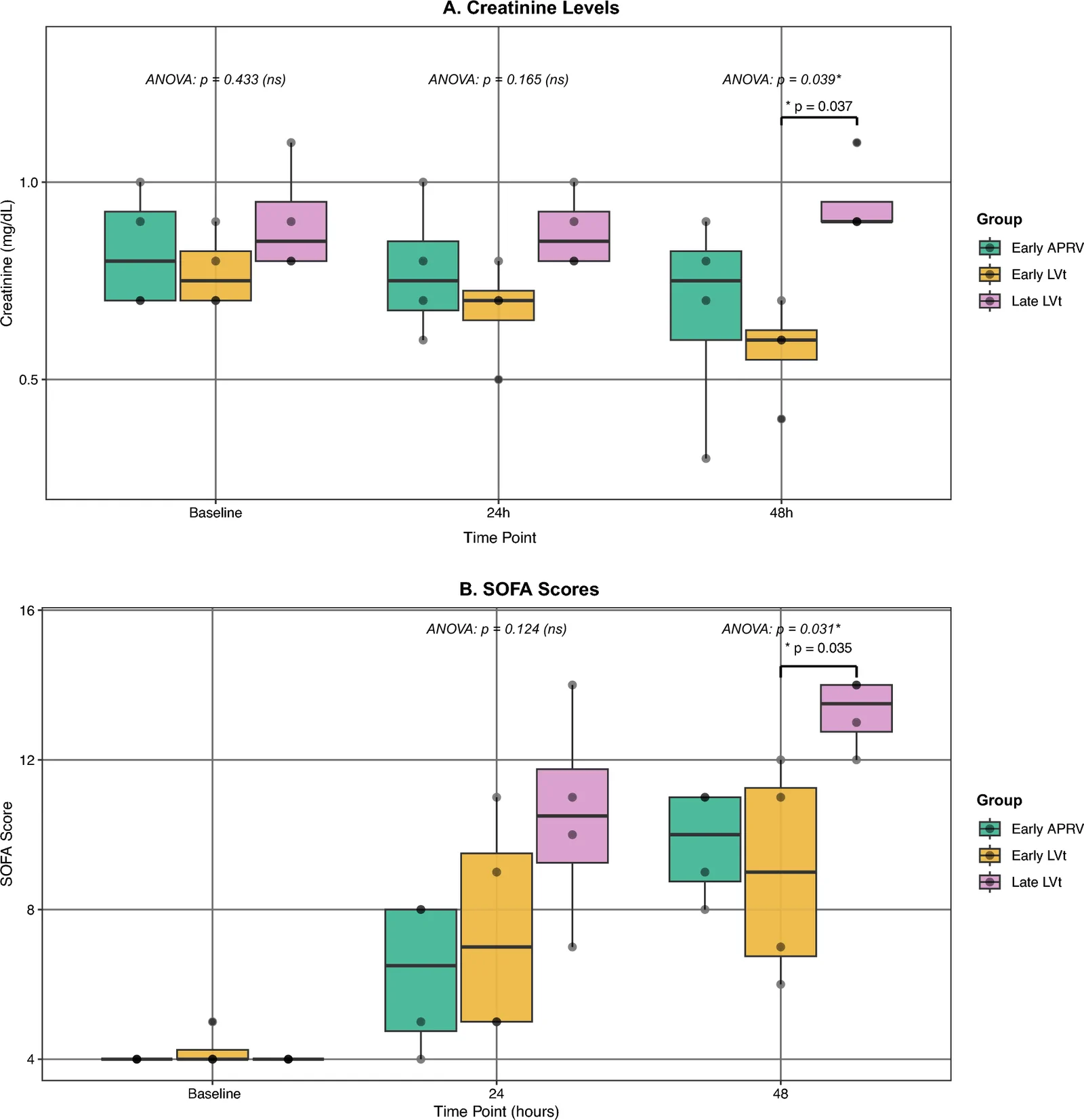
**A**) Creatinine levels (mg/dL) and (**B**) sequential organ failure assessment (SOFA) scores at baseline, 24 h, and 48 h among the three injury groups (APRV—green, Early LVt—orange, and Late LVt – pink. Median represented by black line within each box. The bottom of the box marks the first quartile (25th percentile) and the top marks the third quartile (75th percentile) and individual data points are represented by small circles

There was a significant difference in creatinine across the injury groups at the 48-h time point, with the Late LVt group having a higher creatinine level (0.97±0.07mg/dL) than the Early LVt group (0.58±0.06mg/dL; p<0.05) but similar to the Early APRV group (0.80±0.06mg/dL; p>0.05; Fig. 2A). There was a significant difference in SOFA scores between the Late LVt (13.3±0.5) and Early LVt (9.0±1.5) groups at the 48-h time point (p<0.05; Fig. 2B). Taken together, this suggests that despite the standardized MAP targets, our Late LVt group had a higher degree of occult tissue hypoperfusion and worse shock leading to worsened end organ function.

### Quantitative histology

On quantitative histology, Late and Early LVt were found to have significantly greater alveolar wall thickening as compared with Early APRV and the Naïve animal (p<0.05), with Late LVt having the most significant degree of alveolar wall thickening (p<0.05; Table 1). Early LVt and Naïve animals had the least capillary congestion (p<0.05), but Late LVt and Naïve animals had the least blood in the air compartment (p<0.05). All experimental animals showed increased fibrin deposition compared with naïve animals, consistent with the model’s inflammatory basis (p < 0.05).

**Table 1:** Quantitative histology comparing the Naïve, Late low tidal volume (LVt), Early LVt, and Airway Pressure Release Ventilation (APRV) groups

	**Naïve**	**Late LVt**	**Early LVt**	**APRV**	
Micro-atelectasis	0.00±0.00	0.05±0.05	0.10±0.08	0.00±0.00	P=0.3682
Fibrin Deposition	0.15±0.06*	0.83±0.06*	0.85±0.13*	0.93±0.17*	P<0.0001
Blood in Air Compartment	0.08±0.045. §	0.25±0.089	0.78±0.10*	0.43±0.12’§	P<0.0001
Capillary Congestion	0.35±0.25*	1.75±0.15*	0.75±0.13	0.90±0.16*	P<0.0001
Thickened Alveolar Walls	0.00±0.00§	1.82+0.21*	1.10±0.18*	0.20±0.06.§	P<0.0001

Reported P-values after repeated measures ANOVA. * = p<0.05 vs Naïve; ¶ = p<0.05 vs Late LVt; § = p<0.05 vs Early LVt; † = p<0.05 vs APRV; ° = p<0.05 vs all others after post hoc Tukey’s test for differences between groups

### Principal component analysis of unbiased proteomics

**Fig. 3:**
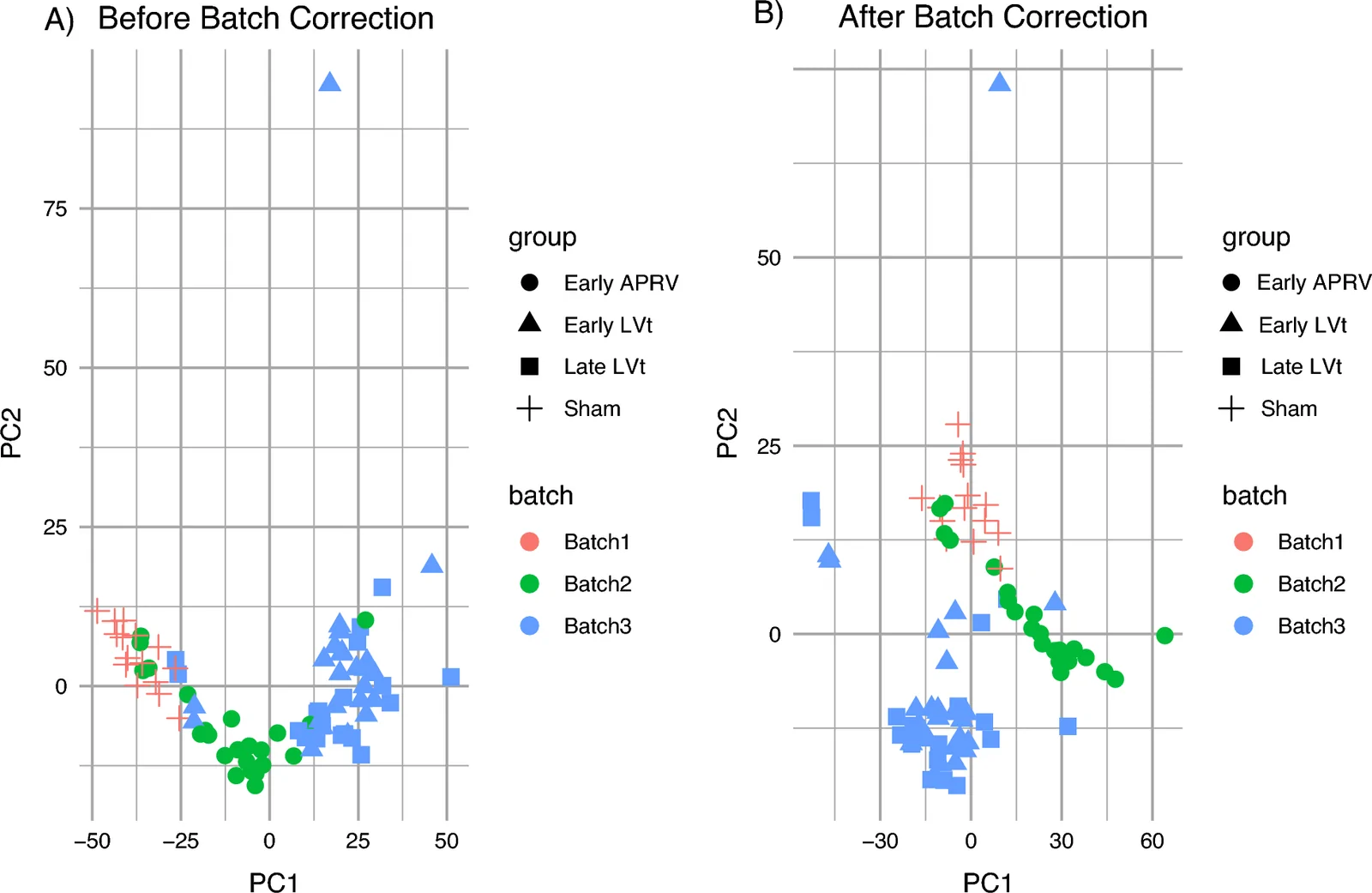
Principal component analysis (PCA) of specimens before (**A**) and after (**B**) batch correction. Each sample is denoted by a shape (group) and color (batch)

There were 2,727 detected proteins across 82 specimens representing four groups (Sham, Early APRV, Early LVT, and Late LVT) at multiple time points (baseline, 6, 12, 24, 36, and 48 h). After filtering out proteins with more than 50% missing values, 1,991 proteins remained for further analysis. PCA analysis of all samples before and after batch correction was performed, to confirm a good result (Fig. 3). We found that the samples were initially strongly clustered by batch, but after batch correction using T0 specimens, this was no longer the case. The batch corrected values were then used for all subsequent analyses. To further quantify the effect of batch, we calculated batch associated variance across all time points before (PERMANOVA R^2^ = 0.28) and after batch correction (PERMANOVA R^2^ = 0.18).

**Fig. 4:**
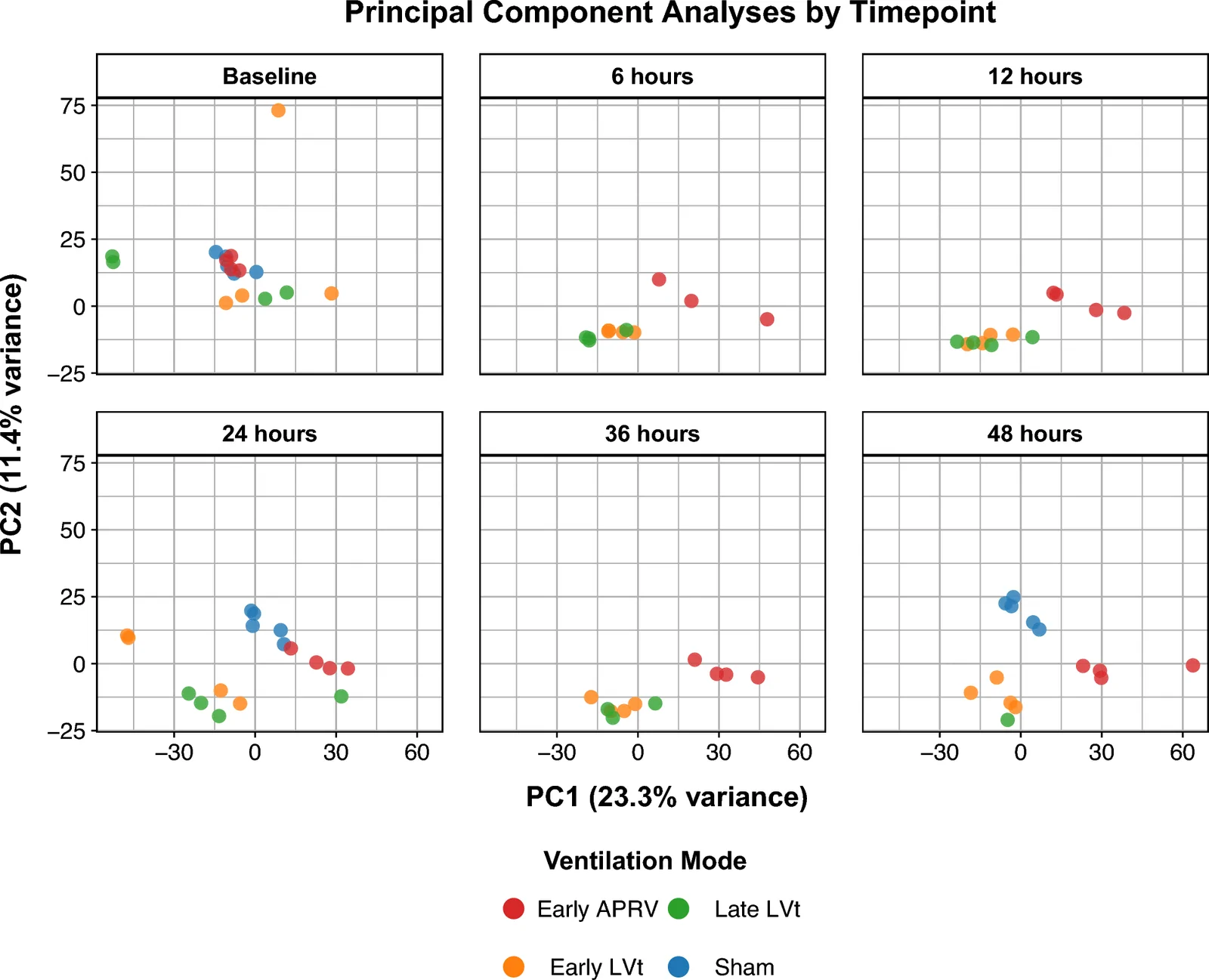
Principal component analysis of each specimen at baseline, 6, 12, 24, 36, and 48 h, showing that the groups were all close together in PCA space at baseline but then slowly separated into three distinct groups by 48 h. The three final groups were Sham animals, the Early low tidal volume (LVt) and one remaining Late LVt animal, and the Early APRV animals

**Fig. 5:**
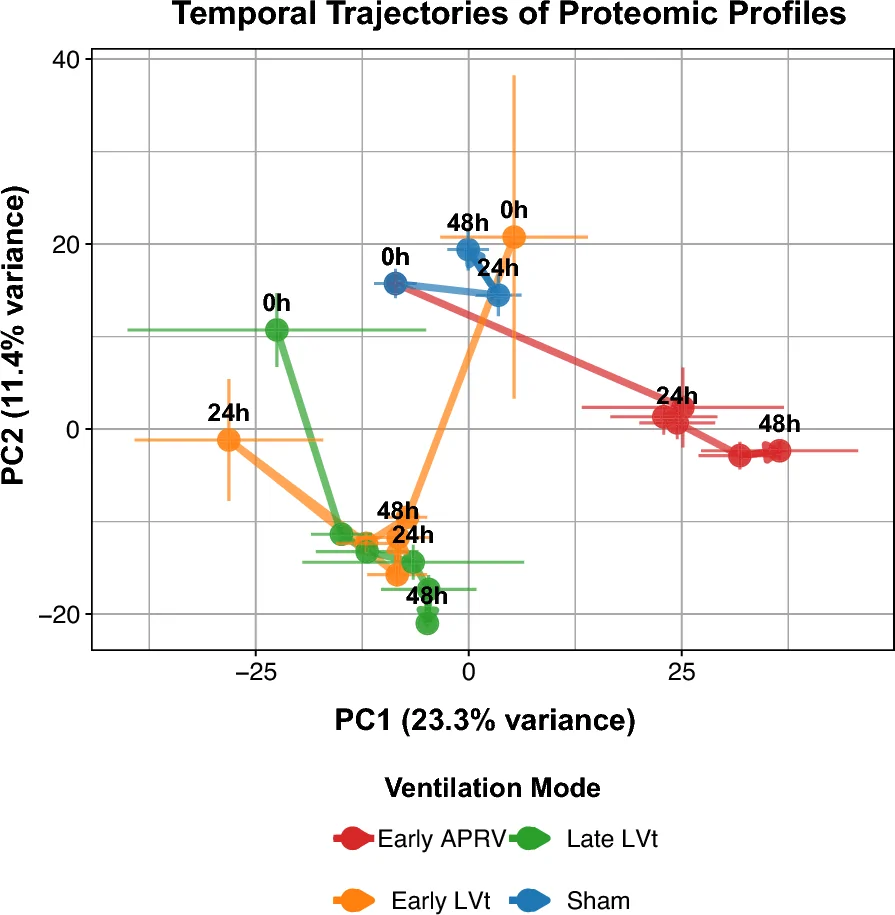
Principal component centroid trajectories over time by group, showing initial close grouping of samples in PCA space. The samples diverge over time into three primary groups. The first group consists of sham animals; the second, Early low tidal volume (LVt) and late LVt animals; and the third, Early APRV animals

PCA was performed and facetted by time point for ease of interpretation. At baseline, samples were clustered closely together in PCA space, except for an outlier in the Early LVt group (Fig. 4). Over the first 24 h, the specimens began to separate into two groups: one comprising the Early and Late LVt animals, and the other containing the Early APRV and sham animals. By 48 h, this separation was more pronounced with clear grouping by experimental condition. The temporal progression of the groups through PCA space was evaluated using centroids (Fig. 5). This again confirmed that groups were initially grouped together in PCA space and then diverged. As expected, the sham animals did not diverge appreciably from baseline. The Late and Early LVt groups were initially grouped closely but distinguished by 48 h, though this distinction is limited by the fact that only one animal in the Late LVt group survived to 48 h. The separation between sham, Early LVt/Late LVt, and Early APRV clearly demonstrates the significant effect of ventilator mode on the plasma proteome. These data suggest that in subjects with sepsis-associated ARDS, the choice of ventilator mode could have systemic impacts that either worsen or improve overall organ dysfunction. We next sought to determine which proteins and pathways were most significantly differentially expressed between the groups.

### Differential protein expression and pathway analysis

**Fig. 6:**
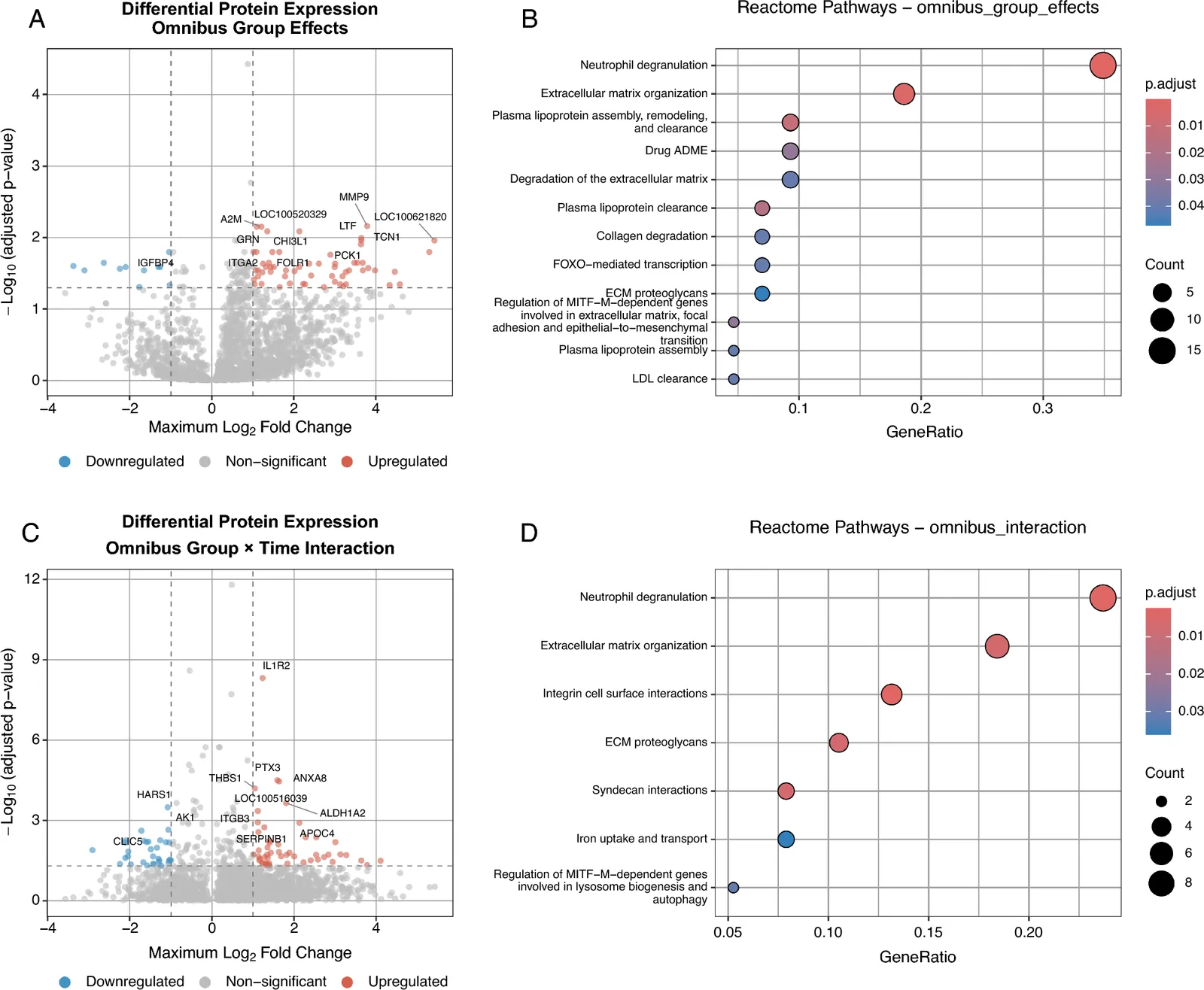
**A**) Volcano plot showing the differential expression of proteins in the group effects model. The top differentially expressed proteins are labeled. **B**) Reactome pathway analysis of the differentially expressed proteins in the group effect model. Most of the implicated pathways are involved in the immune response or tissue remodeling. **C**) Volcano plot showing the differential expression of proteins in the group x time interaction mixed effects model. The top differentially expressed proteins are labeled. **D**) Reactome pathway analysis of the differentially expressed proteins in the group x time interaction model. Most of the implicated pathways are involved in immune responses or tissue remodeling

Mixed-effects modeling was used to identify the pattern of differential expression across all groups and determine the group effect (Fig. 6A). This analysis revealed 125 differentially expressed proteins. Enriched pathways in the group effect model included neutrophil degranulation and several pathways involved in extracellular matrix maintenance and degradation (Fig. 6B). The pattern of differential protein expression across all groups was then examined using a group effect × time interaction term. 198 differentially expressed proteins were identified (Fig. 6C), and pathway analysis again revealed enrichment for neutrophil degranulation and extracellular matrix maintenance and degradation (Fig. 6D). Mixed-effects modeling was used to conduct groupwise comparisons for all possible group combinations. Using this method, a wide range of differentially expressed proteins was found from a low of 25 in the Early LVt vs. Late LVt comparison to a high of 1,194 in the Late LVt vs Early APRV comparison (Supplemental Figs. 3A-89A). All pairwise comparisons that included Early APRV yielded a high number of differentially expressed proteins, consistent with our PCA results, which showed that most Early APRV animals clustered together but were far from other groups in PCA space. Pathway analyses of all differentially expressed proteins were performed across all pairwise comparisons (Supplemental Figs. 3B-8B). The pathway analysis for the Early LVt vs Late LVt comparisons was challenging to interpret due to the low number of differentially expressed proteins between the groups. Most comparisons were enriched in innate immune pathways, including interleukin signaling and neutrophil degranulation. Comparisons of pairs that included Early APRV also tended to include enrichment in macronutrient and cellular metabolic pathways.

### Protein–protein interaction networks

**Fig. 7:**
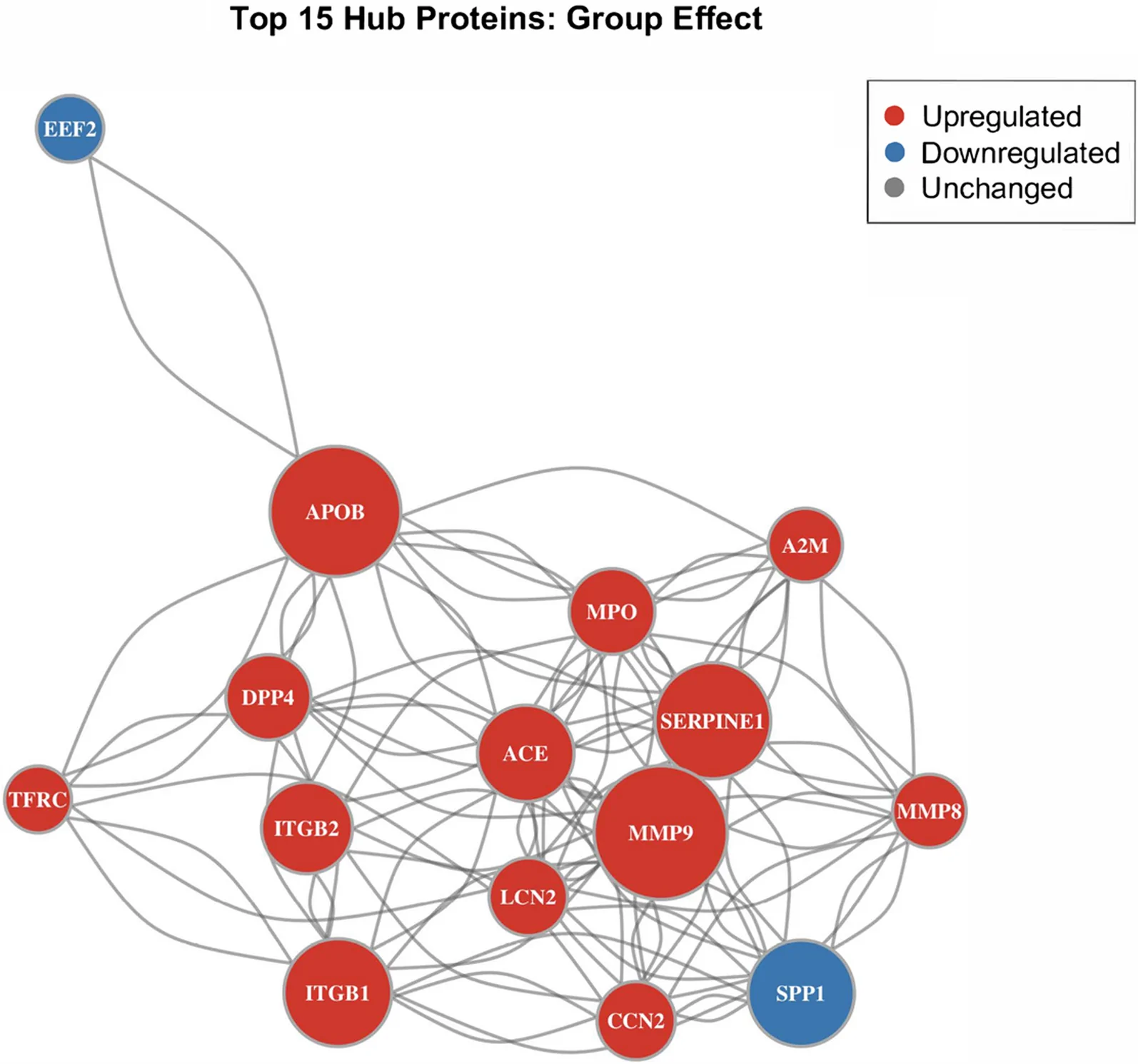
A protein–protein interaction map showing the top 15 hub proteins identified in the group effects model. The size of the circle represents a node’s centrality in the network. The color indicates the direction of change: red for upregulation and blue for downregulation

**Fig. 8:**
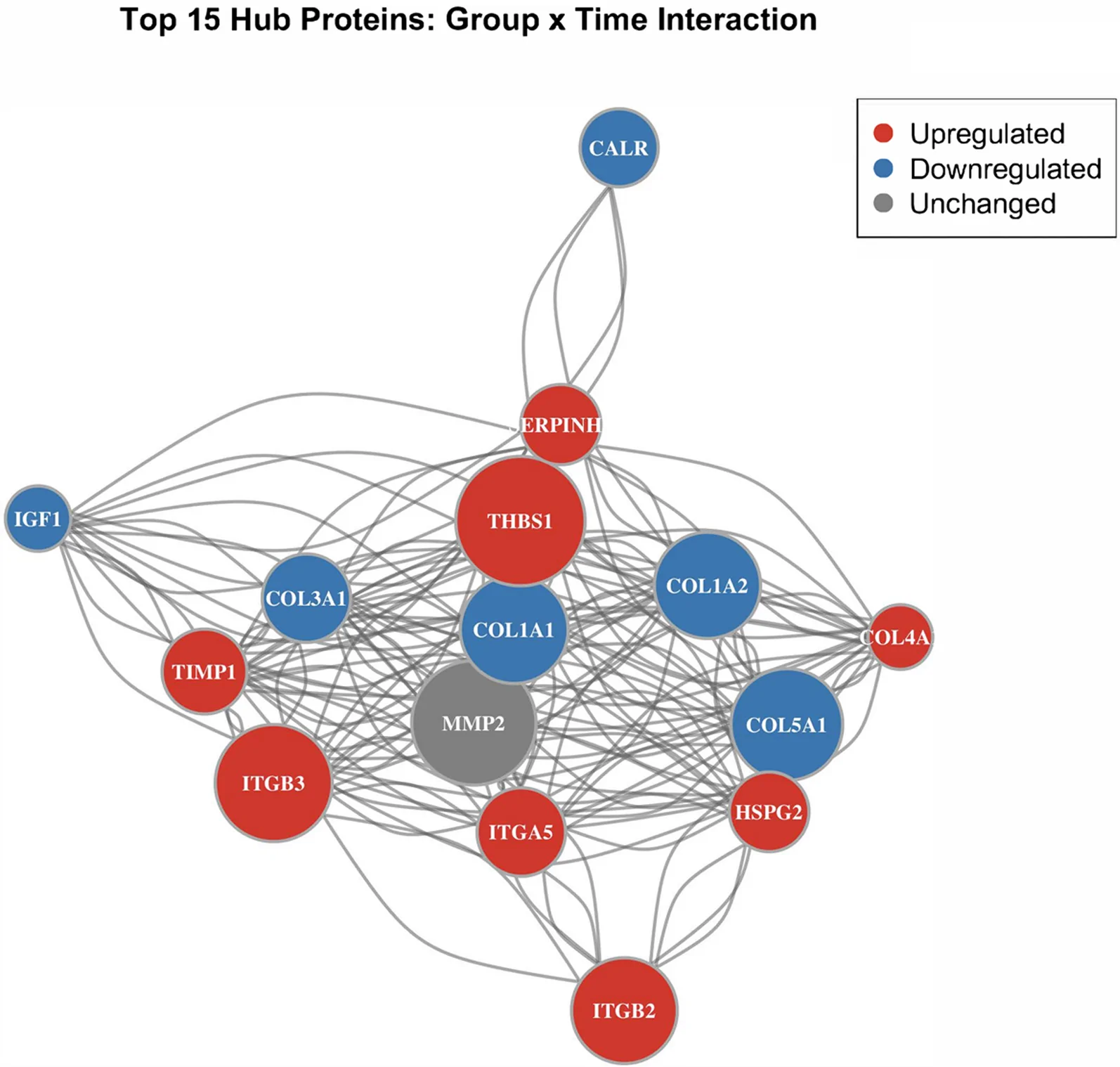
A protein–protein interaction map showing the top 15 hub proteins in the group x time interaction model. The size of the circle represents a node’s centrality in the network. The color indicates the direction of change: red for upregulation, blue for downregulation, and grey for unchanged

Protein–protein interaction networks were developed for the overall group effect, the group x time interaction, and each pairwise comparison. The protein–protein interaction network in the group effect analysis was significant for several proteins involved in the inflammatory response and antiproteases (Fig. 7). The group effect x time interaction network was more skewed towards proteins essential in the extracellular matrix and the unfolded protein response (Fig. 8). The results of the other pairwise comparisons are presented in Supplemental Figs. 9–14. These results were largely consistent with what was observed in the group effect and the group × time interaction, except for the Early LVt vs. Late LVt comparison, which had few proteins in the network due to the low number of differentially expressed proteins.

We then explored the clinical significance of the top five hub proteins identified in the group effect and group effect x time PPI networks by correlating each with lactate and SOFA score. In the group effects model, the correlation between the top five hub proteins with lactate were weak (Table 2), with matrix metalloproteinase 9 (MMP-9) having the strongest correlation (Spearman ⍴ 0.35, p < 0.05). In contrast, the correlations with SOFA score were improved with both MMP-9 and SERPINE1 having strong correlations with SOFA score (Spearman ⍴ 0.59 and 0.68 respectively and p < 0.001 for both). In the group effects x time interaction model, the top 5 identified hub proteins again correlated best with SOFA score (Table 3). Matrix metalloproteinase−2 (MMP-2) and Integrin Subunit Beta 3 (ITGB3) had the most significant correlation with SOFA score (Spearman ⍴ 0.76 and − 0.67 respectively and p < 0.001 for both). In contrast, of the top 5 hub proteins correlated poorly with lactate with the strongest being ITGB3 (Spearman ⍴ − 0.23 but not statistically significant).

**Table 2:** Group effect hub proteins

Protein	Log₂ FC	Lactate ρ	SOFA ρ
MMP9	3.79**	0.35*	0.59***
APOB	0.86*	-0.03	-0.18
SERPINE1	2.42*	0.28*	0.68***
ITGB1	0.64*	0.28*	-0.07
SPP1	-0.63*	0.15	0.23

The correlation (Spearman ρ) of the top five hub proteins identified in the group effects model with lactate and sequential organ failure assessement (SOFA) score. Log2 FC = maximum absolute log2 fold change from omnibus mixed-effects model. Lactate and SOFA correlations are Spearman rank correlations across all available animal timepoint observations. *p.adj < 0.05, **p.adj < 0.01, ***p.adj < 0.001 (Benjamini–Hochberg corrected)

**Table 3:** Group effect×time interaction hub proteins

Protein	Log₂ FC	Lactate ρ	SOFA ρ
THBS1	1.04***	0.05	0.47*
MMP2	-0.16***	0.17	0.76***
ITGB3	1.12**	-0.23	-0.67***
COL5A1	-0.44***	0.20	-0.09
COL1A1	-0.56***	0.05	0.22

The correlation (Spearman ρ) of the top five hub proteins identified in the group effect x time interaction model with lactate and sequential organ failure assessement (SOFA) score. Log2 FC = maximum absolute log2 fold change from omnibus mixed-effects model. Lactate and SOFA correlations are Spearman rank correlations across all available animal timepoint observations. *p.adj < 0.05, **p.adj < 0.01, ***p.adj < 0.001 (Benjamini–Hochberg corrected)

### Differential expression and pathway analysis at T36

**Fig. 9:**
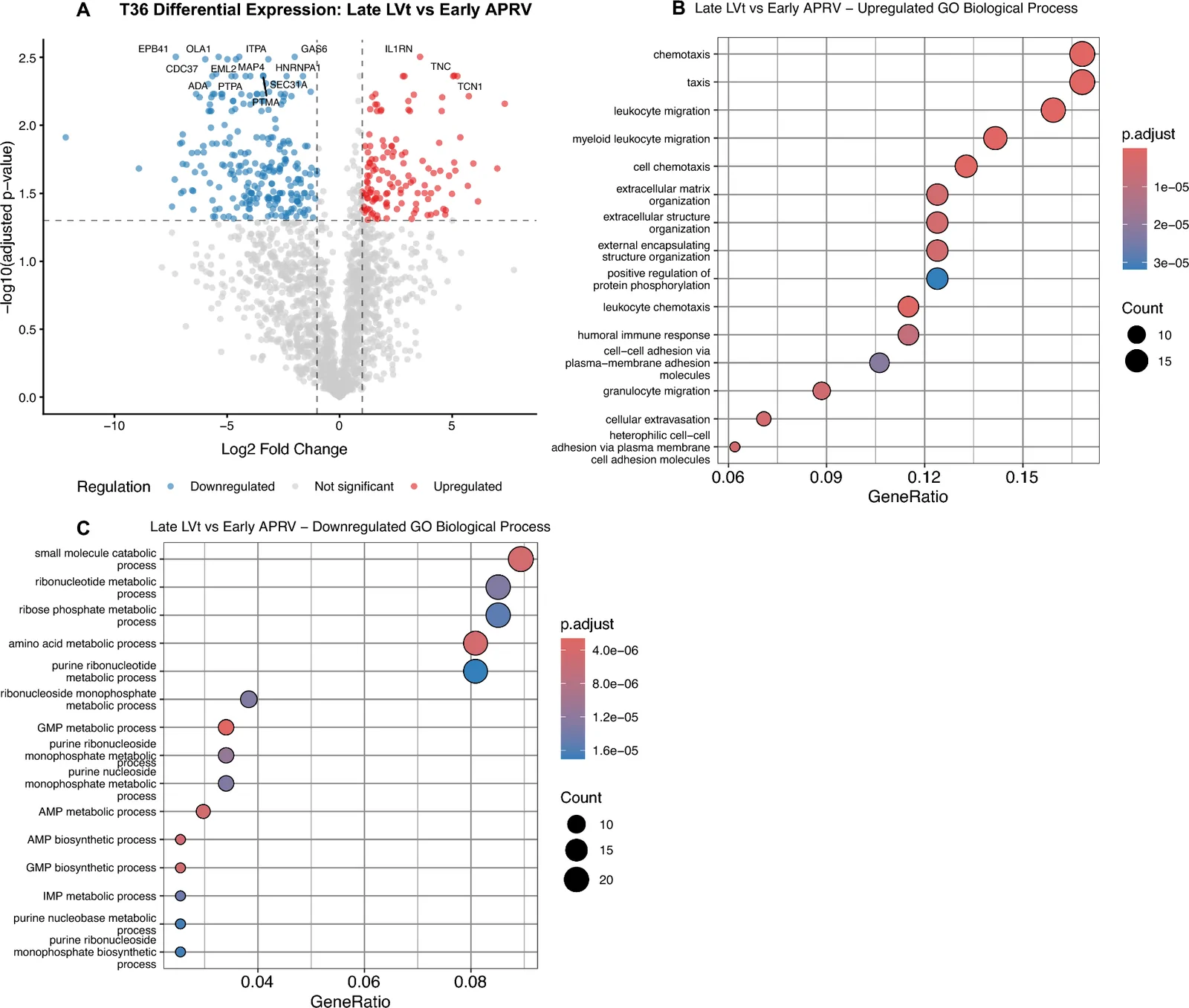
**A**) Volcano plot showing the differential expression of proteins between the late low tidal volume (Late LVt) and early airway pressure release ventilation (Early APRV) groups at the 36-h time point. The top differentially expressed proteins are labeled. **B**) GO biological process pathway analysis of the up-regulated proteins between Late LVT vs. APRV groups at the 36-h time point. Most pathways were directly involved in leukocyte migration or activation. **C**) GO biological process pathway analysis of the downregulated proteins between Late LVt vs. Early APRV groups at the 36-h time point. Most pathways were related to various homeostatic metabolic processes

**Fig. 10:**
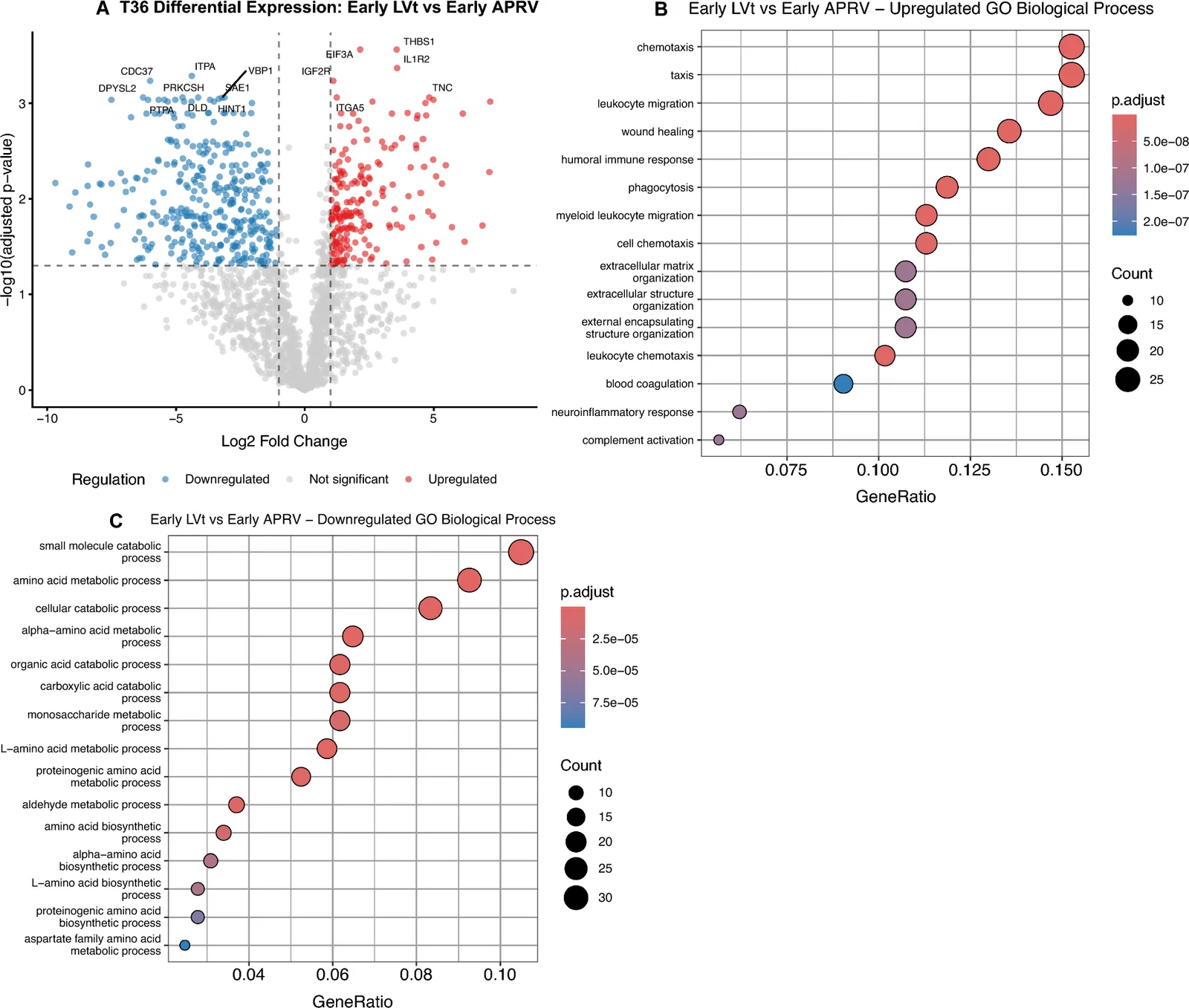
**A**) Volcano plot showing the differential expression of proteins between the early low tidal volume (Early LVt) and early airway pressure release ventilation (Early APRV) groups at the 36-h time point. The top differentially expressed proteins are labeled. **B**) GO biological process pathway analysis of the upregulated proteins between Early LVt vs. Early APRV groups at the 36-h time point. Most pathways were involved in immune cell migration or activation. **C**) GO biological process pathway analysis of the downregulated proteins between the Early LVt vs. Early APRV groups at the 36-h time point. Most of the processes were related to hemostatic metabolic pathways

We compared the plasma proteome between Late LVt vs. Early APRV, Late LVt vs. Early LVt, and Early LVt vs. Early APRV at the T36 time point. The T36 time point was selected as a primary sensitivity analysis because only one Late LVt animal was alive at T48. The goal was to assess the proteome state at the end of the experiment and test the hypothesis that inflammation in the Late LVt group would be higher with longer exposure to the injury. Consistent with the PCA results showing that Late LVt and Early LVt were closely grouped at the end of the experiment, there were no differentially expressed proteins between Late LVt and Early LVt using mixed-effects modeling. 379 differentially expressed proteins between Late LVt vs. Early APRV were identified (Fig. 9A). We further divided by direction of the logFC and were left with 129 upregulated proteins and 250 downregulated proteins. Pathway analyses on the up- and downregulated proteins (Fig. 9B and C) found that many immune and inflammatory pathways were upregulated, while metabolic pathways were downregulated. The Early LVt vs. Early APRV comparison found 551 differentially expressed proteins, with 352 downregulated and 199 upregulated (Fig. 10A). Pathway analyses demonstrated that the upregulated pathways were primarily involved in immune and inflammatory responses (Fig. 10B). In contrast, the downregulated pathways were primarily involved in metabolic and tissue remodeling processes (Fig. 10C).

## Discussion

In this exploratory study, we tested the hypothesis that in our porcine model of sepsis-associated ARDS, animals managed with sustained higher mean airway pressure (Early APRV) would demonstrate less systemic inflammation and organ dysfunction than those managed with relatively lower mean airway pressure (Early LVt) and those with VILI (Late LVt). We found that the three injury groups experienced clinical findings consistent with lung injury and sepsis, including vasopressor-dependent hypotension, bacteremia, and decreasing pulmonary compliance with increasing ventilator pressures required to maintain oxygenation. We showed that the plasma proteomes of the experimental groups differed most significantly in proteins involved in the immune and inflammatory responses. We also showed that the differences in inflammatory pathways between the groups were associated with relevant clinical outcomes, including increasing lung injury severity, lactic acidosis, and SOFA scores.

Sepsis and ARDS are two quintessential ICU syndromes that share many of the same features, including a dysfunctional immune response and heterogeneous underlying biology [4]. Additionally, the two syndromes often occur together, with sepsis being one of the most common triggers for ARDS [4]. While ARDS mortality has remained stubbornly high [43], patients with sepsis-associated ARDS are at a particular risk for morbidity and mortality with increased disease severity and poorer recovery [44]. Less well studied is whether VILI worsens systemic inflammation and organ dysfunction in sepsis. VILI is common in ARDS patients with increasing support requirements, causing mechanical and inflammatory damage that results in the systemic release of inflammatory mediators such as interleukin-1 (IL-1), IL-6, and tumor necrosis factor alpha (TNF-a) [12, 45]. These cytokines are also implicated in the pathophysiology of the dysregulated immune response that defines sepsis [46]. Thus, worsening VILI could augment systemic inflammation and cytokine release, further exacerbating the organ dysfunction associated with sepsis. Our Late LVt group was used to simulate the effects of VILI and our results showing that it was the most inflamed group by the end of the experiment support the theory that VILI is associated with increased levels of inflammatory mediators in the plasma. However, our data are not sufficient to determine if the presence of VILI or differences in the strategy of ventilation alone, are causally related to these proteomic changes.

Surprisingly, we found that the Early LVt group had a similar proteome to that of the Late LVt group, although it was less inflamed. This similarity is likely due to the use of the ARDSnet PEEP/F_i_O_2_ table to set the PEEP in both groups and suggests that the critical physiologic difference between our groups was likely the degree of atelectasis between the Early and Late LVt groups and the early APRV groups. This finding highlights an important consideration regarding the mechanisms of lung injury: atelectrauma versus volutrauma. The Early APRV group had the highest mean Vt at the end of the study (13.2 ml/kg) and lower levels of inflammation at T36 than either Early or Late LVt group. This suggests that atelectrauma may be equally or more harmful than volutrauma, which is supported by other studies investigating time- and pressure-based mechanical ventilation modes [47]. It also bears consideration that a larger tidal volume applied preemptively to a set of open and homogeneous alveoli may not be causing overdistension at the alveolar level [48]. Atelectrauma is defined as the repeated opening and collapse of atelectatic alveoli with each respiratory cycle [49]. APRV is a mode of ventilation that has been shown to potentially improve outcomes in ARDS in both animals and humans [24, 50]. APRV is defined by an inspiratory level of continuous positive airway pressure that alternates with brief expiratory releases. The premise of the mode is to stabilize the lung at a higher mean airway pressure and prevent de-recruitment to reduce atelectrauma [29, 51]. On histology, the Early APRV group demonstrated decreased atelectasis, which provides further evidence that the degree of atelectasis may have contributed to the proteomic differences detected between Early APRV and the LVt groups.

Another important consideration when interpreting our data is the fact that we used an abdominal sepsis model resulting in increased intraabdominal pressure (IAP). Pathologically elevated IAP is classified as intra-abdominal hypertension (IAH) at an IAP ≥ 12 mmHg and abdominal compartment syndrome (ACS) occurs at an IAP ≥ 20 mmHg when associated with new organ dysfunction [52]. Increased IAP modifies pulmonary mechanics by displacing the diaphragm caudally and decreasing the compliance of the respiratory system [53, 54]. These alterations can be significant, with pulmonary compliance decreasing significantly above an IAP of 16 [54, 55]. This results in increased peak inspiratory and plateau pressures [54, 55]. The effect of IAP on pulmonary mechanics may partially explain some of our results. By the end of our experiment, the Early LVt group had intraabdominal hypertension (IAP ≥ 12) and our Early APRV and Late LVt groups had abdominal compartment syndrome (IAP ≥ 20). This could explain why driving pressure was not significantly different between our groups at the end of the experiment as the driving pressure required may have been a function of IAP as opposed to a marker of decreased compliance from lung disease. There is also experimental evidence that optimizing mean airway pressure and potentially increasing tidal volume might reduce atelectasis and systemic inflammation in ARDS with concomitantly elevated IAP [54, 56]. This may explain why our Early APRV group had lower systemic inflammatory signatures at 36 h compared with our Late and Early LVt groups despite having a much higher tidal volume.

Clinically significant differences in pulmonary and extra-pulmonary organ dysfunction were also evident between the groups. The Late LVt group had a lower P/F ratio compared with the Sham and Early APRV groups at the end of the experiment. The Late LVt group also had a significantly higher creatinine and SOFA score than the Early LVt group at the end of the experiment. The differences in the degree of pulmonary and extrapulmonary organ injury provides further evidence to support the theory that the differences observed in the plasma proteome may translate to meaningful clinical outcomes. Taken together, our data suggest that worsening VILI and increased atelectasis could be associated with end-organ dysfunction by way of dysregulated inflammation, although our data are insufficient to determine causality. Even in the absence of infection, mechanical ventilation can upregulate the toll-like receptor 4 (TLR4) signaling pathway, launching an inflammatory cascade similar to that observed in sepsis [57]. Furthermore, pathologic systemic inflammation from VILI has been associated with increased gut permeability in animal models [57].

Future directions following this work should include identifying the most significant proteins that contribute to the pathologic synergism between sepsis, VILI, and extrapulmonary organ injury and correlate this with human data. Proteins identified in our model that are corroborated with human data represent a potentially high-yield target for therapeutic intervention.

Our study has several limitations. The proteomics experiments were designed post hoc to generate hypotheses regarding the mechanisms underlying the observed clinical changes between experimental groups that had been studied in batches. Previously collected data were assessed to avoid unnecessary use of additional animals and replicative experiments. We performed baseline anchored batch correction to minimize batch effects. This strategy was chosen to minimize the dilution of true biological differences as our treatment groups were not balanced across batches. Despite this, the potential for bias due to batch effects remains and our results should be interpreted as hypothesis generating rather than conclusive. An additional limitation is the lack of histologic data for the sham group, which served as the comparison group for the proteomic analysis. However, the sham group did not have an injury induced and did not require increased FiO_2_ or PEEP and had normal lung compliance throughout the study period. Though the sham animals were not at risk for developing lung injury and did not develop clinical acute lung injury, the lack of histologic confirmation is a weakness.

Another limitation of this work is the small sample size of the experimental groups. Since our goal was to generate preliminary data we did not intend to power the study to show causality and our findings should be interpreted with caution until further confirmation. Additionally, the fact that two of our animals in the Late LVt group died before the end of our experiment introduces the possibility of survivor bias. To address this, we conducted a sensitivity analysis at the T36 timepoint as this was the last time point all animals were alive. The results at the T36 time point were consistent with our main results in showing that our Early APRV group had a proteome that was distinct and less inflamed compared to our Early and Late LVt groups. Other limitations of this work include the relatively short time frame of the experiment compared to the typical ICU course of a septic shock patient, the use of the TCAV method with APRV which may not represent the way APRV is utilized in every institution, and the inherent limitations of extrapolating animal data to humans.

## Conclusion

In summary, we found that a strategy of early high sustained mean airway pressure (APRV) was associated with decreased systemic inflammatory signatures compared with conventional low tidal volume ventilation in a porcine model of abdominal sepsis-associated ARDS. Given the small sample size, differential survival between groups, and the inability to separate ventilation mode from mean airway pressure and recruitment strategy, these findings are hypothesis-generating and are not directly translatable to clinical care.

## Supplementary Information

Additional file 1.

Additional file 2: Supplemental Figure 1. A) The level of positive end expiratory pressure (PEEP) in cmH_2_O over time, comparing the sham group to late low tidal volume (LVt), early LVt, and early airway pressure release ventilation (APRV) groups. B) The plateau pressure (cmH_2_O) over time comparing the sham, late LVt, early LVt, and early APRV groups. The plateau pressures were measured directly on the ventilator using an inspiratory hold maneuver. C) The driving pressure (cmH_2_O) over time comparing sham, late LVt, early LVt, and early APRV groups. Driving pressure was calculated as the plateau pressure minus the PEEP at each time point. D) Tidal volume in milliliters per kilogram (ml/kg) over time comparing the sham, late LVt, early LVt, and early APRV groups. E) The arterial P_a_CO_2_ (mmHg) over time comparing the sham, late LVt, early LVt, and early APRV groups.

Additional file 3: Supplemental Figure 2. A) Mean arterial pressure (MAP) over time in the sham, late low tidal volume (LVt), early LVt, and early airway pressure release ventilation (APRV) groups. MAP was measured directly via an arterial catheter. B) The level of lactate (mmol/L) measured on an arterial blood gas over time in the sham, late LVt, early LVt, and early APRV groups. C) Cumulative dose of norepinephrine (mL) over the course of the experiment in the late LVt, early LVt, and early APRV groups. Of note, the sham group never received norepinephrine and was thus not included in this graph. D) Bladder pressure over time (mmHg) measured via a Foley catheter in the sham, late LVt, early LVt, and early APRV groups.

Additional file 4: Supplemental Figure 3. A) Volcano plot showing the differential expression of proteins in the early airway pressure release (APRV) vs. Sham comparison. The top differentially expressed proteins are labeled. B) Reactome pathway analysis of the differentially expressed proteins in the Early APRV vs. Sham comparison. Most of the implicated pathways are involved in the immune response or metabolism.

Additional file 5: Supplemental Figure 4. A) Volcano plot showing the differential expression of proteins between the early low tidal volume (LVt) vs. early airway pressure release ventilation (APRV) groups. The top differentially expressed proteins are labeled. B) Reactome pathway analysis of the differentially expressed proteins between early LVt and early APRV, with most pathways implicated in the immune response and antigen processing/presentation.

Additional file 6: Supplemental Figure 5. A) Volcano plot showing the differential expression of proteins between the early low tidal volume (LVt) vs. late LVt groups. There were very few differentially expressed proteins between the groups. The top differentially expressed proteins are labeled. B) Reactome pathway analysis of the differentially expressed proteins. Interpretation is limited by the small number of differentially expressed proteins and the few significant pathways.

Additional file 7: Supplemental Figure 6. A) Volcano plot showing the differential expression of proteins between the early low tidal volume (LVt) vs. the sham groups. The top differentially expressed proteins are labeled. B) Reactome pathway analysis of the differentially expressed proteins. The most enriched pathways were involved in the immune response and extracellular matrix maintenance.

Additional file 8: Supplemental Figure 7. A) Volcano plot showing the differential expression of proteins between the late low tidal volume (LVt) vs. early airway pressure release ventilation (APRV) groups. The top differentially expressed proteins are labeled. B) Reactome pathway analysis of the differentially expressed proteins. The most enriched pathways were involved in the immune response and metabolism.

Additional file 9: Supplemental Figure 8. A) Volcano plot showing the differential expression of proteins between the late low tidal volume (LVt) vs. the sham groups. The top differentially expressed proteins are labeled. B) Reactome pathway analysis of the differentially expressed proteins. The most enriched pathways were involved in the immune response and extracellular matrix maintenance.

Additional file 10: Supplemental Figure 9. Protein-protein interaction network of the top 15 hub proteins in the early airway pressure release ventilation (APRV) vs. sham comparison. The size of the circle represents the degree of centrality in the network, and the direction of regulation is denoted by color.

Additional file 11: Supplemental Figure 10. Protein-protein interaction network of the top 15 hub proteins in the early low tidal volume (LVt) vs. early airway pressure release ventilation (APRV) comparison. The size of the circle represents the degree of centrality in the network, and the direction of regulation is denoted by color.

Additional file 12: Supplemental Figure 11. Protein-protein interaction network of the top 15 hub proteins in the early low tidal volume (LVt) vs. late LVt comparison. The size of the circle represents the degree of centrality in the network, and the direction of regulation is denoted by color.

Additional file 13: Supplemental Figure 12. Protein-protein interaction network of the top 15 hub proteins in the early low tidal volume (LVt) vs. sham comparison. The size of the circle represents the degree of centrality in the network, and the direction of regulation is denoted by color.

Additional file 14: Supplemental Figure 13. Protein-protein interaction network of the top 15 hub proteins in the late low tidal volume (LVt) vs. early airway pressure release ventilation (APRV) comparison. The size of the circle represents the degree of centrality in the network, and the direction of regulation is denoted by color.

Additional file 15: Supplemental Figure 14. Protein-protein interaction network of the top 15 hub proteins in the late low tidal volume (LVt) vs. sham comparison. The size of the circle represents the degree of centrality in the network, and the direction of regulation is denoted by color.

Additional file 16: Supplemental Table 1: Study timeline describing the initial ventilator protocols for each group followed by the ventilator titration parameters used after injury. LV_T_ = low tidal volume, APRV = airway pressure release ventilation, VC = volume control, V_T_ = tidal volume, PEEP = peak end expiratory pressure, RR = respiratory rate, FiO_2_ = fraction of inspired oxygen, SpO_2_ = oxygen saturation, ARDS = acute respiratory distress syndrome, P/F = P_a_O_2_/FiO_2_ ratio, Pplat = plateau pressure, P_High_ = pressure high, P_Low_ = pressure low, T_High_ = time high, T_Low_ = time low, PEFR = peak expiratory flow rate, EEFR = end expiratory flow rate, BL = baseline.

## Data Availability

The datasets used and analyzed during the current study are available from the corresponding author on reasonable request.

## Abbreviations

ARDS: Acute respiratory distress syndrome
VILI: Ventilator-induced lung injury
LVt: Low tidal volume
APRV: Airway pressure release ventilation
TCAV: Time controlled adaptive ventilation
Vt: Tidal volume
PEEP: Positive end expiratory pressure
VC: Volume control
RR: Respiratory rate
FiO_2_: Fraction of inspired oxygen
ABG: Arterial blood gas
IAP: Intra-abdominal pressure
BL: Baseline
IV: Intravenous
MAP: Mean arterial pressure
SpO_2_: Oxygen saturation
Pplat: Plateau pressure
PEFR: Peak expiratory flow rate
EEFR: End expiratory flow rate
SOFA: Sequential organ failure assessment
PCA: Principal component analysis
PPI: Protein–protein interaction
P/F: Partial pressure of arterial oxygen to fraction of inhaled oxygen ratio
TC-PEEP: Time-controlled PEEP
IL-1: Interleukin-1
IL-6: Interleukin-6
TNF-a: Tumor necrosis factor-a
TLR4: Toll-like receptor 4
