# Supplementary material for: The impact of ventilation mode on systemic inflammation and organ dysfunction in a porcine model of abdominal sepsis-associated acute respiratory distress syndrome
Source: Intensive Care Med Exp. 2026 Oct 10;14:133. doi: 10.1186/s40635-026-00985-3 (PMC13653911; doi:10.1186/s40635-026-00985-3)
Supplement: Supplementary file 7 [file 40635_2026_985_MOESM16_ESM.pdf]

Supplementary Table 1

Experimental Timeline and Ventilator Management by Treatment Group

Sham · Late LV_T · Early LV_T · Early APRV

GROUP	PROTOCOL (BL → T0)	T0 (Injury) → T48	
Sham	**Peri-Procedural VC**<br>V_T 10 mL/kg · PEEP 5<br>RR 12 · FiO_2 100%	**Volume Control: Sham Laparotomy → T48**<br>V_T 10 mL/kg · PEEP 5 cmH_2O · RR 12<br>*Titration: FiO_2 adjusted to maintain SpO_2 ≥ 90%. No other ventilator changes.*	
Late LV_T	**Peri-Procedural VC**<br>V_T 10 mL/kg · PEEP 5<br>RR 12 · FiO_2 100%	**VC: Conventional V_T**<br>V_T 10 mL/kg · PEEP 5<br>RR 12 · FiO_2 21%<br>*No titration. Maintained until Berlin ARDS criteria met.* →	**VC: Low V_T (when P/F < 300 → T48)**<br>V_T 6 mL/kg · PEEP ≥ 5 cmH_2O<br>*Titration: PEEP & FiO_2 per ARDSNet PEEP/FiO_2 table. RR titrated for pH > 7.2. V_T adjusted to keep P_{plat} < 30 cmH_2O or until min 4 mL/kg.*
Early LV_T	**Peri-Procedural VC**<br>V_T 10 mL/kg · PEEP 5<br>RR 12 · FiO_2 100%	**VC: Low V_T (Immediate at T0 → T48)**<br>V_T 6 mL/kg · PEEP 5 cmH_2O · RR 12 · FiO_2 21%<br>*Titration: PEEP & FiO_2 per ARDSNet low PEEP/high FiO_2 table. RR titrated to maintain pH > 7.2. V_T adjusted to keep P_{plat} < 30 cmH_2O or until min 4 mL/kg.*	
Early APRV	**Peri-Procedural VC**<br>V_T 10 mL/kg · PEEP 5<br>RR 12 · FiO_2 100%	**APRV / TCAV: Initial Settings**<br>• **P_{High}**: Set at the plateau pressure (P_{plat}) obtained during the VC setting used for BL measurements (16–22 cmH_2O).<br>• **T_{High}**: 4.0–4.5 s, set to occupy approximately 90% of the total ventilator cycle time.<br>• **P_{Low}**: Set at 0 cmH_2O to minimize expiratory resistance and maximize the peak expiratory flow rate (PEFR).<br>• **T_{Low}**: Set to achieve an EEFR:PEFR of 75% (0.32–0.37 s).	**APRV / TCAV: Titration**<br>P_{High}, P_{Low}, T_{High}, T_{Low}, and FiO_2 were titrated throughout the study according to pulmonary parameters, PaO_2, and $PaCO_2$.

Timeline: BL — T0 (Injury) — T48
