# Supplementary material for: The impact of ventilation mode on systemic inflammation and organ dysfunction in a porcine model of abdominal sepsis-associated acute respiratory distress syndrome
Source: Intensive Care Med Exp. 2026 Oct 10;14:133. doi: 10.1186/s40635-026-00985-3 (PMC13653911; doi:10.1186/s40635-026-00985-3)

Supplemental Figure 7 Late LVt vs. Early APRV

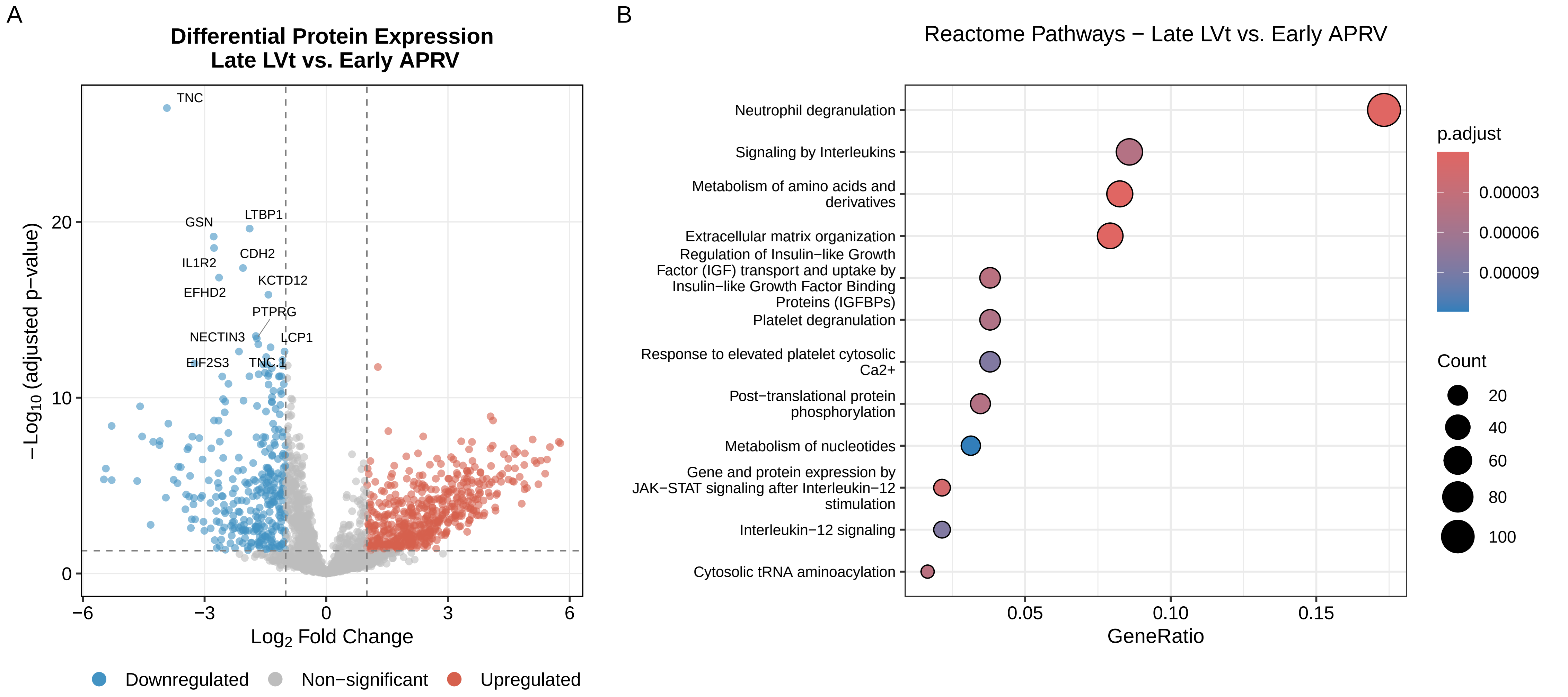

Supplement: Supplementary file 14 [file 40635_2026_985_MOESM8_ESM.pdf]
