# Supplementary material for: The impact of ventilation mode on systemic inflammation and organ dysfunction in a porcine model of abdominal sepsis-associated acute respiratory distress syndrome
Source: Intensive Care Med Exp. 2026 Oct 10;14:133. doi: 10.1186/s40635-026-00985-3 (PMC13653911; doi:10.1186/s40635-026-00985-3)
Supplement: Supplementary file 16 [file 40635_2026_985_MOESM1_ESM.docx]

Supplementary Material – Methods

**Housing, Husbandry, and Monitoring**

Pigs were transported from a USDA-approved farm to the Department of Laboratory Animal Resources (DLAR) at SUNY Upstate Medical University. They were provided chow and water ad lib until the night before the procedure, at which time they were made NPO. Pigs were housed in pens that were cleaned twice daily. They were provided enrichment activities including marshmallows, popsicles, and balls. The DLAR staff would regularly play with the pigs, and pigs were allowed to cohabitate with other pigs. They were allowed a 7-day acclimatization period prior to experiments.

All animal experiments were conducted at SUNY Upstate Medical University. Animals were pretreated with atropine (0.04 mg/mL), tiletamine hydrochloride and zolazepam (5mg/kg) and xylazine (2mg/kg) intramuscularly. An IV catheter was placed into an ear vein for continuous infusion of ketamine (9mg/mL) / xylazine (0.009mg/mL) and continued through a central venous line, once placed. Animals were continuously monitored for anesthesia adequacy ensuring a Stage III, Plane 2 level of anesthesia. All animals were humanely euthanized at the study end point (48 hours after injury) or if they were found to no longer have a survivable injury, as determined by refractory septic shock (unresponsive to fluids and maximal vasopressor support), or refractory acute respiratory distress syndrome (persistent PaO_2_:FiO_2_ < 50 mmHg despite maximal ventilatory support) with a pentobarbital (150 mg/kg) overdose.
